# Mother's Satisfaction towards Childbirth Care at Public Health Centers in Bench-Maji Zone, Ethiopia: A Facility-Based Cross-Sectional Study

**DOI:** 10.1155/2020/6746459

**Published:** 2020-07-01

**Authors:** Shewangizaw Hailemariam, Amare Genetu, Ermiyas Sahile

**Affiliations:** ^1^Department of Midwifery, College of Health Science, Mizan-Tepi University, Mizan-Teferi, Ethiopia; ^2^Department of Midwifery, College of Health Science, Kotebe Metropolitan University, Addis Ababa, Ethiopia

## Abstract

**Background:**

Assessing the level of maternal satisfaction towards maternal health care services has a paramount importance in improving the service quality and enhancing service utilization. Hence, the aim of this study was to assess maternal satisfaction towards childbirth care and its determinants at public health facilities in Bench-Maji Zone, Ethiopia.

**Methods:**

A facility-based cross-sectional study was conducted from May 20, 2018, to July 11, 2018 in Bench-Maji Zone, Ethiopia. A total of 845 mothers were selected by employing a systematic random sampling technique. Data were collected using a pretested and structured questionnaire. Satisfaction was measured by the five-point Likert scale from very dissatisfied (1) to very satisfied (5). Data were entered in to Epi data version 3.1 and analyzed using SPSS version 20. A *P* value < 0.05 was considered to declare statistical significance.

**Result:**

About 506 (63.25%) of the mothers were satisfied by the overall care provided during childbirth. Factors associated with mothers' satisfaction with childbirth care includes attending no formal education [AOR = 3.69; 95% CI (1.99, 7.91)], rural residency [AOR = 2.63; 95% CI (1.43, 5.80)], perceived measure taken to assure privacy [AOR = 3.56; 95% CI (1.25, 7.41)], and attending antenatal care [AOR = 6.23; 95% CI (3.42, 12.87)].

**Conclusion:**

The overall satisfaction of mothers with childbirth care in public health centers of Bench-Maji Zone is low when compared with other studies. Hence, understanding mothers' expectations, assuring privacy, and enhancing antenatal care attendance might improve maternal satisfaction with childbirth care.

## 1. Background

Mothers' satisfaction with childbirth care is a multidimensional construct comprising satisfaction with technical aspects, environmental aspects, and communication and interpersonal aspects [1]. The assessment of mothers' satisfaction adds an important “consumer” perspective to evaluations. Feedback from mothers can influence the whole quality improvement agenda within the institution or organization [2]. Mothers who perceive the quality of care as good, are more likely to visit it again, thereby increasing demand for the service [3, 4]. Maternal health service utilization and neonatal outcome can be considerably enhanced by improving the quality of facility childbirth care.

Globally, low-middle-income countries continue to account for almost all of the pregnancy-related mortalities that are largely preventable through adequate utilization of essential maternal health care services such as skilled birth assistance (SBA) and antenatal care (ANC) [5]. Studies suggest that utilization of SBA and ANC is highly influenced by mothers' satisfaction [6]. Hence, the World Health Organization (WHO) recommend mothers' service satisfaction be assessed to improve the quality and effectiveness of health care [7].

It is a well-established fact that maternal satisfaction on the provided service could enhance subsequent utilization and compliance with medical treatments [8–10]. However, there is a limited study regarding the extent of mothers' satisfaction with delivery service in Ethiopia. Hence, this study will provide evidence regarding mothers' satisfaction with childbirth care and its determinants.

## 2. Methods

### 2.1. Study Area and Design

Facility-based cross-sectional study was conducted from May 20 to July 11, 2018, in Bench-Maji Zone which is located 561 km away from Addis Ababa, the capital city of Ethiopia, in Southwest direction with an estimated population of 829,493 out of which 418,213 are women; 129,500 are children under five; and 26,462 are under 1 year. The zone has 39 functional public health centers. Additionally, there are 182 functional health posts and 9 under construction, one university, and one health science college. The zone has 182 midwives, 329 nurses, and 476 health extension workers [11].

### 2.2. Source and Study Population

Source population were all mothers who gave birth in public health centers of Bench-Maji Zone, and the study population were all mothers who gave birth in selected public health centers of Bench-Maji Zone from May 20, 2018, to July 11, 2018.

### 2.3. Sample Size and Sampling Procedure

During the calculation of the sample size, the proportion of mothers satisfied with delivery service in Bench–Maji Zone was assumed to be 50%. Based on the assumption that the actual sample size for the study was determined using a single-population proportion formula with a marginal error of 5% and 95% confidence level, the sample size became 384. Considering design effect, the sample size was multiplied by 2 and a 10% nonresponse rate was added and the final sample size became 845.

A two-stage sampling technique was employed to select study participants. At the first stage, twelve public health centers (30% of the public health centers) were selected from a total of 40 public health centers found in Bench-Maji Zone. Second, the total sample size was proportionally allocated to each of the selected public health centers based on the average number of delivery in the most recent quarterly report of each health centers. Finally, after calculating “*K*” for each public health centers, the study participants were selected by using a systematic random sampling technique.

### 2.4. Data Collection Tool and Procedure

Exit interview was made by using a structured tool having four sections: the first section contains sociodemographic characteristics of the mother, the second section contains obstetric characteristics of the mother, the third was about mother's perception of health care service, and the forth section contains satisfaction-related questions. Client satisfaction measuring instrument was adapted from Ethiopian Ministry of Health and contextualized to labor and delivery setting. The satisfaction items were grouped into three categories: technical aspects, environmental aspects, and communication and interpersonal aspects. Each components of satisfaction were measured by 6 items. Overall mothers' satisfaction was measured by 18 items each having a five-point Likert scale from very dissatisfied (1) to very satisfied (5). The overall satisfaction scale had an internal consistency or reliability score of 0.83. Data were collected by twelve midwives after having 3 days of training about the data collection process and the purpose of the study. During data collection, the data was checked for completeness and consistency of information. The overall data collection process was supervised by three public health officers. The local language Amharic was used to collect the data.

### 2.5. Operational Definition

Client overall satisfaction and component-wise client satisfaction were dichotomized into satisfied and dissatisfied depending on the threshold which was computed using the demarcation threshold formula: ((total highest score − total lowest score)/2) + total lowest score [10].

### 2.6. Data Processing and Analysis

Data were checked for completeness, edited, cleaned, coded, and entered in to EpiData version 3.1 and then was exported to SPSS version 20 for analysis. In the descriptive statistics, frequencies, proportion, and mean was calculated and the results of the analysis were presented in text and tables. Binary logistic regression was carried out to assess the association of different independent variables with the dependent variable. Independent variables having *P* < 0.25 on the binary logistic regression analysis were considered candidates for the final multivariate logistic regression analysis. Multivariate logistic regression analysis was carried out to identify factors having statistically significant associations with mothers' overall satisfaction with childbirth care.

### 2.7. Data Quality Assurance

The questionnaire prepared in English was translated into Amharic and then back translated into English to ensure consistency. The questionnaire was pretested on 5% of the actual sample size in Maji district. There was supervision on a daily basis and checking on 10% of the collected questionnaire. Finally, error reports were checked after entry to EpiData using each case code.

### 2.8. Ethical Consideration

Ethical clearance was obtained from Mizan-Tepi University. Permission was also obtained from Bench-Maji zone Health department and health centers as well. Efforts were made to keep confidentiality of the data; all participants were reassured that no personal identifier will be used. Verbal informed consent was sought from all study participants.

## 3. Result

### 3.1. Sociodemographic Characteristics of the Respondents'

A total of 94.7%, *n* = 800, mothers willingly responded to the interviewer-administered structured questionnaire. The mean age of the respondents' was 24.7 (±3.26) years. The minimum and the maximum age of the respondents' were 18 and 39, respectively. Of the total respondents, 404 (50.5%) had no formal education and 344 (43%) were housewives. The majority 729 (91.1%) of respondents were married, over 330 (41.25%) of respondents were Protestant Christians, and 305 (38%) of the respondents were Orthodox Christians. Twenty-six percent of the respondents reside in urban areas (Table 1).

### 3.2. Obstetrics and Other Maternal Factors

Regarding obstetrics characteristics, 723 (90.4%) had antenatal care follow-up, 324 (40.5%) were parity I, 44 (5.5%) claimed to had abortion, and 73 (9.1%) were unwanted pregnancy, and 320 (40%) mothers had previous facility-based childbirth experience. Five hundred sixty-seven (70.9%) of the mothers were prepared for labor and delivery, 704 (88%) of the deliveries were attended by female service provider, 136 (17%) of the deliveries end up in immediate maternal complication, and 35 (4.4%) of the deliveries became still birth as shown in (Table 2).

### 3.3. Maternal Perception of Health Care Service

With respects to maternal perception of facility service availability, 734 (91.8%) of the mothers mentioned the presence of waiting area, and 678 (84.8%) mentioned the presence of toilet in the facility. Five hundred thrifty-four (66.8%) of the mothers claimed the presence of an attendant throughout delivery, 312 (39%) of the mothers claimed that they have waited more than 15 minute to be seen by the care provider, 659 (82.4%) of the mothers were informed about the wellbeing of their baby following examination, and care providers measure taken to assure privacy were regarded as “good” by 659 (82.4%) of the mothers, summarized in (Table 3).

### 3.4. Maternal Satisfaction with Delivery Care Services

Regarding the three dimensions of satisfaction (component wise satisfaction), technical aspects had the highest median scores followed by communication and interpersonal aspects and environmental aspects. Pertaining to the overall mothers' satisfaction, 506 (63.25%) of the mothers were satisfied with the overall service provided. Mothers were asked whether they would come again and recommend others to utilize facility-based childbirth care service; more than 88% of the mothers mention that they would come again and 95% of mothers claimed to recommend others to deliver at the public health centers (Table 4).

### 3.5. Factors Associated with Maternal Satisfaction with Delivery Care

After adjusting for “fetal outcome,” “sex of the attendants,” “presence of maternal complication,” “being prepared for labor and delivery,” “client informed about the wellbeing of the baby,” and “status of the pregnancy,” four variables, namely, educational status, residence, measure taken to assure privacy, and attending ANC were found to be significant in the final multivariate logistic regression analysis as shown in (Table 5). Mothers with no formal education and mothers with secondary school were 3.6 and 1.6 times, respectively, more likely to be satisfied by the overall delivery service provided than mothers with educational status diploma and above (AOR = 3.69; 95% CI (1.99, 7.91) and AOR = 1.60; 95% CI (1.34-2.96), respectively). Mothers from rural residence were AOR = 2.63 (95% CI (1.43, 5.80)) times more likely to be satisfied by the overall delivery service provided than those from urban residence. Mothers who regarded measure taken to assure privacy as “good” were AOR = 3.56 (95% CI (1.25, 7.41)) times more likely to be satisfied by the overall delivery service provided than their counterpart. Mothers who fully attended all the WHO-recommended ANC visits were AOR = 6.23 (95% CI (3.42, 12.87)) more likely to be satisfied by the overall delivery service provided than those who did not.

## 4. Discussion

The present study assessed the level of mothers' satisfaction with delivery service and the associated factors; the study revealed that 63.25%, 95% CI (60.08%-66.41%), of mothers were satisfied with the overall delivery services provided, and this finding is much lower than studies undertaken in Assela Hospital (80.7%), Wolaita zone (82.9%), Debre Markos town (81.7%), and Omo Nada District, Jimma Zone (77%) [12–15]. Nevertheless, it is higher than studies conducted in Gondar teaching hospital; Northwest Ethiopia (31.3%); Nairobi, Kenya (56%); and Sri Lanka (48%) [16–18]. The reason for this discrepancy might be due to socioeconomic characteristic of the study population and the type of facility studied and could also be due to the methodological difference in which we used a threshhold demarcation formula to set the cutoff point for satisfaction.

When it comes to component-wise satisfaction, technical aspects had the highest median scores followed by communication and interpersonal aspects and environmental aspects. In contrary to this finding, the study conducted in Sri Lanka claimed a lower level of satisfaction with technical aspects compared to any another components [18]. This disparity could be due to study setting difference in which our study was conducted among health centers; higher satisfaction with technical aspect at health centers than hospitals might imply work fatigue as a health care provider at a hospital level attends to many deliveries in a day than one working at health-center level because of high client flow. Environmental aspect had the lowest median score than any other components, and this is consistent with the study undertaken in South Ethiopia and contradicts with the study conducted in India and Bangladesh—in which a relatively high proportion of clients are satisfied with environmental aspect [19, 20]. This could be explained as hospitals are relatively more equipped with facility infrastructures like toilet, water, and electricity compared with health centers.

The current study evidenced a reciprocal relationship between educational status and mother satisfaction; mothers with secondary school and lower educational status were more likely to be satisfied with delivery service than those with diploma and above educational status. This finding is in agreement with the study conducted in Arba Minch, Ethiopia, Tanzania, Ghana, and Zambia [21–24]. Higher satisfaction in low literacy setting might attribute to expectation difference that more educated mothers could have higher expectation since they are aware of the care standards and client rights than less educated mothers.

Mothers from rural residence were AOR = 2.63 (95% CI (1.43, 5.80)) times more likely to be satisfied by the overall delivery service provided as compared to mothers from urban residence. This finding is substantiated by other studies conducted in Addis Abeba, Ethiopia, Tshwane, South Africa, and Brazil, where mothers coming from rural residence were more satisfied than those from urban residence [25–27]. This might be due to the difference in media exposure; mothers from rural residence might have poor media exposure regarding delivery service and have less service expectations compared to their urban counterpart.

The other most important predictor of client satisfaction in this study was mother's perceived measure taken to assure her piracy. Those mothers who regarded measure taken to assure their privacy as “good” were AOR = 3.56 (95% CI (1.25, 7.41)) times more likely to be satisfied than their counterpart. Similar finding was suggested by studies conducted in Southern Ethiopia, Northwest Ethiopia, Malawi, and Zambia [25, 28–30]. This could be explained as privacy is a key requirement for a women in the delivery process. A sense of shame is attached to it; hence, inadequate privacy during the procedure increases women's discomfort and diminishes their satisfaction levels.

Mothers who fully attended all the WHO-recommended ANC visits were AOR = 6.23 (95% CI (3.42, 12.87)) times more likely to be satisfied by the overall delivery service provided than those who did not attend ANC. This finding is similar with study conducted in Gamo Gofa Zone, Ethiopia [21]. This might be for the fact that ANC users get the necessary preventive care and are less likely to encounter pregnancy complications. Moreover, mothers who had attended ANC would have prepared for labor and delivery and may not expect everything from the health facility.

## 5. Conclusion

The overall satisfaction of mothers with childbirth care in public health centers of Bench-Maji Zone is low when compared with other studies. Mothers' educational status, residency, measure taken to assure privacy, and ANC attendance were significantly associated with mothers' satisfaction with overall delivery service provided. Thus, the Ethiopian Federal Ministry of Health and other relevant stakeholders working on enhancing maternal health service should focus on these determinates to improve maternal satisfaction with childbirth care and ultimately reduce maternal mortality and morbidity in the area.

## Figures and Tables

**Table 1:** The sociodemographic characteristics of mothers who gave birth in selected public health centers of Bench-Maji Zone, Ethiopia. May 2018. *n* = 800.

Variable	Response	Frequency	Percentage	*P* value
Age	<20	50	6.25	
	20-24	269	33.62	0.345
	25-29	328	41.0	0.269
	30-34	104	13	0.540
	>35	49	6.12	0.641
Residence	Urban	212	26.5	
	Rural	588	73.5	0.001
Marital status	Single	71	8.9	
	Married	729	91.1	0.461
Ethnicity	Bench	307	38.37	
	Keffa	202	25.25	0.913
	Amhara	239	29.9	0.822
	Oromo	40	5	0.322
	Tigra	7	0.87	0.252
	Gurage	5	0.62	0.716
Educational status	No formal education	404	50.5	0.001
	Primary school	267	33.37	0.004
	Secondary school	99	12.37	0.002
	Diploma and above	30	3.75	
Religion	Orthodox	305	38	
	Muslim	158	19.75	0.632
	Protestant	330	41.25	0.461
	Catholic	5	0.62	0.357
	Traditional beliefs	2	0.25	__
Occupational status	Government worker	27	3.4	
	Non-government worker	190	23.8	0.376
	Merchant	60	7.5	0.420
	Housewife	344	43	0.652
	Daily laborer	123	15.4	0.516
	Student	56	7	0.441

**Table 2:** The obstetric characteristics of mothers who gave birth in selected public health centers of Bench-Maji Zone, Ethiopia. May 2018. *n* = 800.

Variables	Response	Frequency	Percent	*P* value
Parity	1	324	40.5	
	2-4	397	49.6	0.398
	≥5	79	9.9	0.382
Ever had abortion	Yes	44	5.5	
	No	756	94.5	0.269
Status of pregnancy	Wanted	727	90.9	
	Unwanted	73	9.1	0.149
Ever delivered in health facility	Yes	320	40	
	No	480	60	0.509
Prepared for labor and delivery	Yes	567	70.9	0.071
	No	233	29.1	
Sex of attendant	Female	704	88	0.205
	Male	96	12	
Maternal complication immediately	Yes	136	17	0.187
	No	664	83	
Fetal outcome	Live birth	765	95.6	
	Still birth	35	4.4	0.097
Privacy	Good	513	64.1	
	Poor	287	35.9	0.001
ANC attended	Yes	723	90.4	
	No	77	9.6	0.001

**Table 3:** Maternal perception of health care service among mothers who gave birth in selected public health centers of Bench-Maji Zone, Ethiopia. May 2018. *n* = 800.

Variables	Response	Frequency	Percent	*P* value
Perceived presence of waiting area	Yes	734	91.8	
	No	66	8.2	0.901
Presence of toilet	Yes	678	84.8	
	No	122	15.2	0.551
Care provider measures taken to assure privacy	Good	659	82.4	
	Not good	141	17.6	0.001
Presence of attendant throughout delivery	Yes	534	66.8	
	No	266	33.2	0.641
Mother informed about the baby following examination	Yes	498	62.2	
	No	302	37.8	0.407
Waiting time to be seen by the provider	15 minute	488	61	
	More than 15 minute	312	39	0.312

**Table 4:** Median satisfaction score of the three dimensions of satisfactions among mothers gave birth in selected public health centers of Bench-Maji Zone, Ethiopia. May 2018.

Dimensions	Median
Technical aspects	
Level of satisfaction with pain management	5
Level of satisfaction with support given during labor & delivery	4
Level of satisfaction with care given to newborn	5
Level of satisfaction with time given by providers	4
Level of satisfaction with continuity of care	5
Level of satisfaction with general care received	5
Communication and interpersonal aspects	
Level of satisfaction with explanation before procedures	4
Level of satisfaction with promptness of response	5
Level of satisfaction with listening concern	4
Level of satisfaction with supportiveness of staff	5
Level of satisfaction with allowing socials to be together	5
Level of satisfaction with respect	4
Environmental aspects	
Level of satisfaction with easily getting maternity ward starting from the gate	4
Level of satisfaction with maternity ward's toilets, hand washing and shower.	3
Level of satisfaction with cleanness of the ward	5
Level of satisfaction with privacy	4
Level of satisfaction with infrastructures like electricity, water	4
Level of satisfaction with easily getting maternity ward starting from the gate	5

**Table 5:** Factors significantly associated with overall mothers' satisfaction in multivariate logistic regression, Bench-Maji Zone, Ethiopia. May 2018.

Variables	Level of satisfaction		COR (95% CI)	AOR (95% CI)	*P* value
	Satisfied	Dissatisfied			
	Count (%)	Count (%)			
Educational status					
No formal education	317 (78.46%)	87 (21.53%)	11.97 (5.01-29.43)	3.69 (1.99, 7.91)	0.001
Primary school	137 (51.31%)	130 (48.69%)	3.46 (2.91-7.01)	1.27 (1.03, 1.94)	0.034
Secondary school	45 (45.45%)	54 (54.54%)	2.73 (1.59-6.05)	1.60 (1.34-2.96)	0.012
Diploma and above	7 (23.33%)	23 (76.67%)	1	1	
Residence					
Urban	88 (46.22%)	124 (53.78%)	1	1	
Rural	418 (69.39)	170 (30.61%)	3.47 (1.99-10.14)	2.63 (1.43, 5.80)	0.001
Measure taken to assure privacy					
Good	387 (75.43%)	126 (24.57%)	4.33 (2.77-9.01)	3.56 (1.25, 7.41)	0.001
Poor	119 (41.46%)	168 (58.54%)	1	1	
ANC attended					
Yes	435 (65%)	288 (30%)	1	1	
No	71 (46.75%)	6 (53.24%)	7.83 (5.78-19.43)	6.23 (3.42, 12.87)	0.001
Fetal outcome					
Live birth	476 (62.22)	289 (37.78)	1	1	
Still birth	30 (85.71%)	5 (14.29)	3.64 (1.07-6.03)	1.27 (0.87, 29.11)	0.631
Sex of the attendants					
Female	462 (65.62%)	242 (34.37%)	1	1	
Male	44 (45.83%)	52 (54.16%)	0.44 (0.16-0.71)	0.19 (0.01-1.06)	0.701
Presence of maternal complication					
Yes	75 (55.14%)	61 (44.85%)	0.65 (0.29, 0.9)	0.25 (0.11, 1.09)	0.167
No	437 (65.22%)	233 (34.78%)	1	1	
Being prepared for labor and delivery					
Yes	386 (68.08)	181 (31.92%)	1	1	
No	120 (51.5%)	113 (48.49%)	0.49 (0.06, 1.04)	1.09 (0.24, 29.07)	0.408
Client informed about the wellbeing of the baby					
Yes	314 (63.05%)	184 (36.95%)	0.98 (0.11, 1.52)	0.41 (0.21-2.97)	0.091
No	192 (63.58%)	110 (36.42%)	1	1	
Status of the pregnancy					
Wanted	444 (61.07%)	283 (38.93%)	0.28 (0.18, 0.97)	0.45 (0.31, 2.03)	0.338
Unwanted	62 (84.93%)	11 (15.07%)	1	1	

## Data Availability

The datasets collected and analyzed for the current study is available from the corresponding author and can be obtained upon reasonable request.

## Abbreviation

AOR: Adjusted odd ratio
ANC: Antenatal care
CI: Confidence intervals
SBA: Skilled birth assistance
SPSS: Statistical Package for Social Scientists
WHO: World Health Organization.
